# A dataset of the quality of soybean harvested by mechanization for deep-learning-based monitoring and analysis

**DOI:** 10.1016/j.dib.2023.109833

**Published:** 2023-11-22

**Authors:** Man Chen, Chengqian Jin, Youliang Ni, Tengxiang Yang, Jinshan Xu

**Affiliations:** Nanjing Institute of Agricultural Mechanization, Ministry of Agriculture and Rural Affairs, Nanjing Jiangsu Province, 210014, China

**Keywords:** Soybean, Mechanized harvesting, Quality detection, Image classification, Feature extraction

## Abstract

Deep learning and machine vision technology are widely applied to detect the quality of mechanized soybean harvesting. A clean dataset is the foundation for constructing an online detection learning model for the quality of mechanized harvested soybeans. In pursuit of this objective, we established an image dataset for mechanized harvesting of soybeans. The photos were taken on October 9, 2018, at a soybean experimental field of Liangfeng Grain and Cotton Planting Professional Cooperative in Guanyi District, Liangshan, Shandong, China. The dataset contains 40 soybean images of different qualities. By scaling, rotating, flipping, filtering, and adding noise to enhance the data, we expanded the dataset to 800 frames. The dataset consists of three folders, which store images, label maps, and record files for partitioning the dataset into training, validation, and testing sets. In the initial stages, the author devised an online detection model for soybean crushing rate and impurity rate based on machine vision, and research outcomes affirm the efficacy of this dataset. The dataset can help researchers construct a quality prediction model for mechanized harvested soybeans using deep learning techniques.

Specifications Table

**Table:** 

Subject	Agriculture Engineering, Computer Vision and Pattern Recognition, Artificial Intelligence
Specific subject area	Deep-learning-based image detection and classification of the quality of soybeans harvested by mechanization
Data format	Raw: JPG Conversion: JPG AND PNG Annotation: TXT
Type of data	Image
Data collection	Collected images of mechanized harvesting of soybeans. An industrial camera (1080P(V5610)_PCBA, Midway Vision Technology, Guangzhou, China) was used to capture images of soybeans harvested using a soybean combine harvester. The original image format was JPG, with a size of 1280 * 1024 px. These images of mechanically harvested soybeans with different qualities were taken on October 9, 2018, at the soybean testing field of Liangfeng Grain and Cotton Planting Professional Cooperative in Guanyi District, Liangshan, Shandong, China. The shooting process was supplemented with an LED visual light source, with coordinates of 35.28278° N and 116.54047° E. A total of 40 images were captured. These images were enhanced by scaling, rotating, flipping, filtering, and adding noise, which expanded the dataset to 800 images. We manually annotated the original soybean images, annotated the category and location information using different colors, and saved annotations in PNG format for use by different models. The soybean image dataset was divided into training, validation, and testing sets suitable for deep learning and machine learning. The folder size of the soybean image dataset is 328 MB, and a compressed file in RAR format is provided for easy download.
Data source location	Institution: Liangfeng Grain and Cotton Planting Professional Cooperative City: Liangshan, Shandong Country: China Latitude and longitude (and GPS coordinates, if possible) for collected samples/data: 35. 79241°N and 116. 28097°E, Altitude: 40 msl
Data accessibility	Repository name: Mendeley Data Data identification number: 10.17632/hwdtghttp7.2 Direct URL to data: https://data.mendeley.com/datasets/hwdtghttp7/2
Related research article	

## 1 Value of the Data


 

• The soybean image dataset can help related researchers to develop online quality detection models for mechanized harvested soybeans using deep learning and machine vision algorithms. Moreover, studying the quality of mechanized soybean harvesting can effectively monitor the operational performance of soybean harvesters, help drivers grasp the quality of soybean harvesting in real time, and reduce soybean machine harvesting losses, and ensure soybean machine harvesting quality, and improve harvesting efficiency.
• The soybean image dataset contains images of mechanically harvested soybeans with varying qualities. Firstly, agricultural researchers can utilize this dataset to develop online detection models for soybean crushing rate and impurity rate employing diverse deep learning and machine vision models, thereby offering technical support for the development of related parameter online detection sensors. Secondly, by conducting an analysis of the quality of mechanized soybean harvesting through datasets, soybean harvester production enterprises can enhance the design of machinery, leading them to the development of more efficient equipment. Thirdly, researchers in other fields, such as computer science, image analysis and artificial intelligence, can leverage this dataset to assess the performance of model classifiers.
• We developed the neat and clean image dataset of different mechanized harvesting soybeans. All 40 images were automatically captured using industrial cameras during the mechanized soybean harvesting process and then manually marked by field experts.
• Detecting the number of crushed grains in the dataset can help researchers calculate the crushing rate of mechanized soybean harvesting and understand whether the crushing rate of the soybean harvester meets operational requirements.
• Detecting the amount of impurities in the dataset can help researchers calculate the impurity content of mechanically harvested soybeans and understand whether the impurity content of the soybean harvester meets operational requirements.

## 2 Data Description

The dataset described in this article includes 40 mechanically harvested soybean images of different qualities, and the data were enhanced by scaling, rotating, flipping, filtering, and adding noise, which expanded the dataset to 800 images. All images were obtained with the help of soybean research experts. All labeled image components have been categorized into four distinct groups: intact grains, crushed grains, impurities, and background. Moreover, all original images have a resolution of 96 dpi, a width of 1280 px, and a height of 1024 px. The collected data are shown in Table 1.

**Table 1:** Brief description about the data collection.

No.	Particulars	Description
1	Grain	Soybean
2	Photo Time	October 9, 2018
3	Geographical location	35.79241°N and 116.28097°E., Altitude: 40 msl
4	Climate	Sunny
5	Temperature	16 °C–19 °C

The soybean image dataset contains a total of 40 JPG original images, which collected during the mechanized harvesting of soybeans. The soybean image dataset can be used to detect the quality of mechanized harvested soybeans. For these 40 images, we used LabelMe (Version No. 3.16.7, Massachusetts Institute of Technology, Cambridge, MA, USA) to label the soybean image dataset, to label soybean grains and impurities with polygons, and then assigned labels. We divided the image components into four different categories: background, complete grains, broken grains, and impurities. Then, the data were enhanced by random scaling (0.6–1.2 times), random rotation (−180°–180°), mirroring (horizontal, vertical, horizontal vertical), filtering (Gaussian filtering, mean filtering, median filtering, and bilateral filtering), and noise addition (Gaussian noise, salt and pepper noise, Poisson noise, and Speckle noise), which expanded the set to 800 images, as shown in Table 2.

**Table 2:** Description of the dataset for the mechanized harvesting of soybeans.

Data enhancement	Original	Scale	Rotate	Mirror	Filtering	Noise	All
Num.	40	160	160	120	160	160	800

### 2.1 Component description of mechanized harvest soybean image

**Fig. 1:**
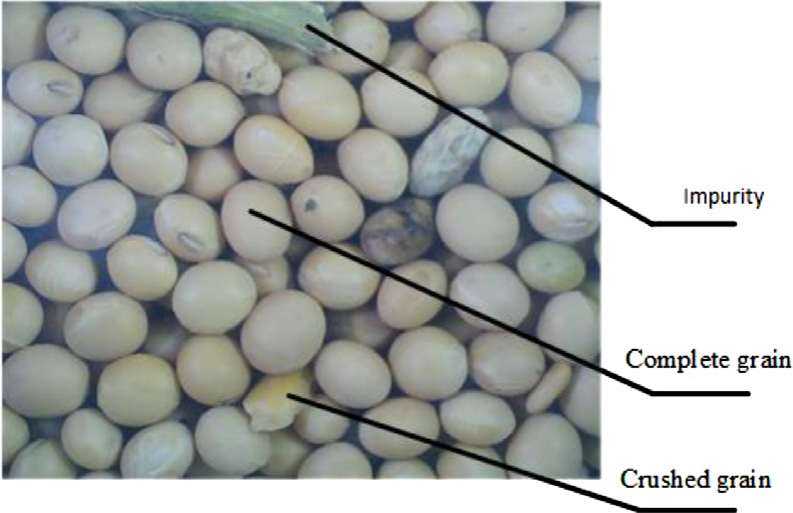
The soybean sample image.

Fig. 1 shows the most common components in soybean samples: intact seeds (i.e. soybean seeds without mechanical damage), broken grains (i.e., grains with skin damage and breakage caused by mechanical harvesting), and impurities, namely of the straw variety (e.g., pods, plant stems, and leaves).

### 2.2 Significance of the dataset

Online monitoring of harvest quality is challenging in mechanized soybean operations. In recent years, researchers have solved this technical challenge through machine vision and deep learning techniques. In contrast to manual detection, online detection of soybean crushing rate and impurity rate based on machine vision and deep learning offers clear advantages. Its exceptional performance is characterized by rapid detection speed, straightforward operation, and reduced human interference. A standard image dataset is the foundation for establishing an online monitoring model for harvest quality. Thus, we constructed a standardized mechanized soybean harvest image dataset. This dataset contains typical forms of intact seeds, crushed grains, and impurities in mechanized soybean harvesting, which is beneficial for researchers to analyze the quality of mechanized soybean harvesting.

To enrich the dataset, we also enhanced the data. In addition, differently from other agricultural datasets, our data come from the process of soybean mechanized harvesting, so they reflect the actual situation of soybean mechanized harvesting. The notable contribution of this dataset is that it provides an opportunity for studying the quality detection of soybeans during mechanized harvesting processes, which can facilitate the detection of changes in soybean quality during mechanized harvesting processes and help drivers detect abnormal harvests promptly, adjust harvester operating parameters, and minimize economic losses to the greatest extent possible.

In summary, the soybean image dataset was manually annotated by field experts to create a usable mechanized soybean quality classification dataset, providing a resource for the development of online detection methods and equipment for soybean harvest quality. In addition, it also provides assistance to grain harvester developers, who can use real images of mechanized soybean production processes for research and then analyze the quality of mechanized harvest based on specific images to obtain real-time harvest crushing rate and impurity rate. Ultimately, through the development of online detection sensors for the quality of soybean harvesting operations, this dataset can provide suggestions for harvester drivers to adjust harvester parameters promptly, helping them improve the efficiency of mechanized harvesting. On the one hand, this dataset can serves as a valuable resource for fundamental research on online detection methods pertaining to the crushing rate and impurity rate in mechanized soybean harvesting; On the other hand, it can aid manufacturers of soybean harvesters in enhancing product quality. Therefore, the dataset has strong practical significance.

### 2.3 Description of dataset folders

**Fig. 2:**
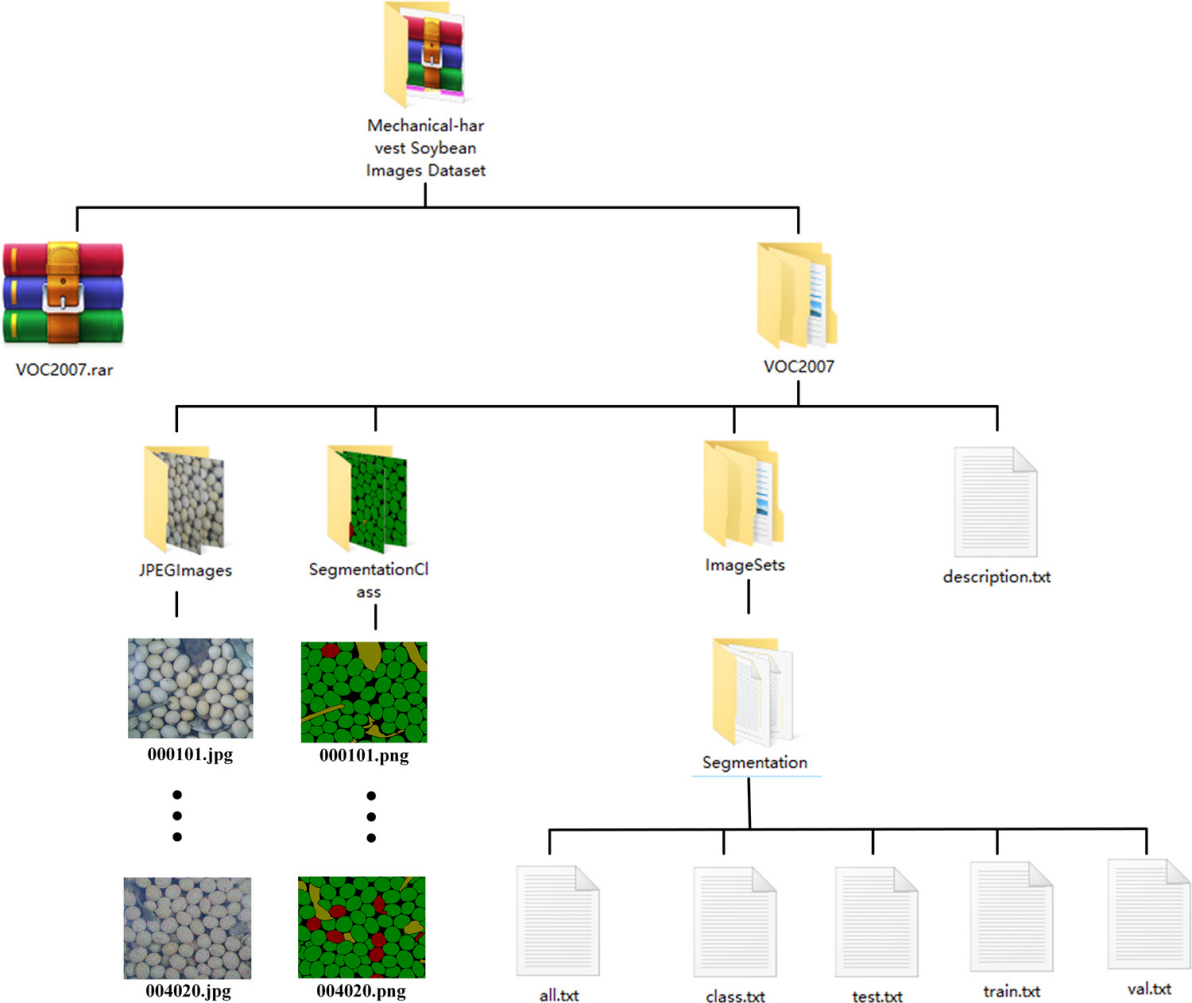
Folder structure.

Following the format of the PASCAL VOC 2007 dataset, we constructed a soybean image dataset by employing 800 mechanically harvested images post data augmentation. These images were segregated into separate folders, with original images and annotated images placed in distinct directories. Subsequently, we partitioned them into training sets, validation sets, and testing sets, preserving the segmentation results in the corresponding TXT files. Table 3 shows a brief description of the dataset. The folder structure is shown in Fig. 2.

**Table 3:** Brief description of the dataset.

No.	Particulars	Description
1	Grain	Soybean
2	Original Image	JPG; 1280∗1024 px; 96 dpi
3	Converted image	JPG and PNG; 1280∗1024 px; 96 dpi
4	Annotation file format	TXT
5	Dataset size	Size of each image: 39–1621 KB JPEGImages folder size: 277 MB SegmentationClass folder size: 50.2 MB ImageSets folder size: 12.6 KB VOC2007 folder size: 327 MB VOC2007.rar folder size: 320 MB

The Mechanical Harvest Soybean Images Dataset directory contains a folder named VOC2007 and a zip file named VOC2007.rar. The VOC2007 folder makes it convenient for researchers to browse and view datasets online, while the VOC2007.rar file can be used to download directly. Researchers only need to download and extract the files to obtain the original soybean image dataset. The roles of the folder and file are shown in Table 4.

**Table 4:** Brief description of the folder/file.

No.	Name	File formats in the folder	Description
1	VOC2007.rar	RAR (in itself)	Conveniently packaged for download
2	VOC2007	–	–
3	JPEGImages folder	JPG	Image files for easy viewing
4	SegmentationClass folder	PNG	Labeled with information such as category and location. The model can be used for training.
5	ImageSets folder	TXT (in Main folder)	It is used to divide the training set, validation set, and test set. It contains file names by which the model can find the corresponding file.

The soybean image dataset's VOC2007 directory comprises three main folders and one TXT file. Within this structure, the JPEGImages folder stores the original images of mechanically harvested soybeans and their augmented counterparts. The SegmentationClass folder stores manually annotated images, while the ImageSets folder maintains image partition ownership information. Additionally, a TXT file explains the description of the dataset, called description.TXT.

#### 2.3.1 JPEGImages folder

This folder contains all 800 images in JPG format. The folder size is 277 MB. The naming convention for images is six Arabic numerals. The first four digits represent the serial number of the field shot image. The last two represent the type of data enhancement, labeled from 1 to 20, where 1 represents the original image, 2–5 represent random scaling, 6–9 represent random rotation, 10–12 represent mirroring, 13–16 represent filtering, and 17–20 represent adding noise. For example, 000,509.JPG indicates that the image was obtained by randomly rotating the soybean image with the sequence number 5, and it is numbered 000,509 among all images.

#### 2.3.2 SegmentationClass folder

The annotation file contains 800 images in PNG format. The folder size is 50.2 MB, and the naming convention is the same as that for the “JPEGImages” folder, which consists of six Arabic numerals and corresponds to the images in the “JPEGImages” folder. Each PNG file contains different labeled components of soybeans, with green representing intact soybean seeds, yellow representing impurities, black representing background, and red representing broken soybean seeds.

#### 2.3.3 ImageSets folder

There is a folder called Segmentation in the folder, which contains five TXT files: all.txt, class.txt, train.txt, val.txt, and test.txt. All.txt contains a random sorting of data numbers for the entire dataset, with a total of 800 rows and one number per row. Train.txt contains the data numbers of randomly selected training datasets, with a total of 560 rows of data, each row containing one number. Val.txt contains the data numbers of randomly selected validation datasets, totaling 120 rows of data, with one number per row. Test.txt contains the data numbers of randomly selected test datasets, totaling 120 rows of data, with one number per row. All numbers correspond to the numbering of the original image. Class.txt explains the classification and annotation rules for the dataset.

In summary, the VOC2007 folder of the soybean image dataset contains training sets, validation sets, and test sets, with 560, 120, and 120 mechanized harvested soybean images, respectively, totaling 800 soybean images.

## 3 Experimental Design, Materials and Methods

### 3.1 Mechanized soybean harvesting image acquisition system

**Fig. 3:**
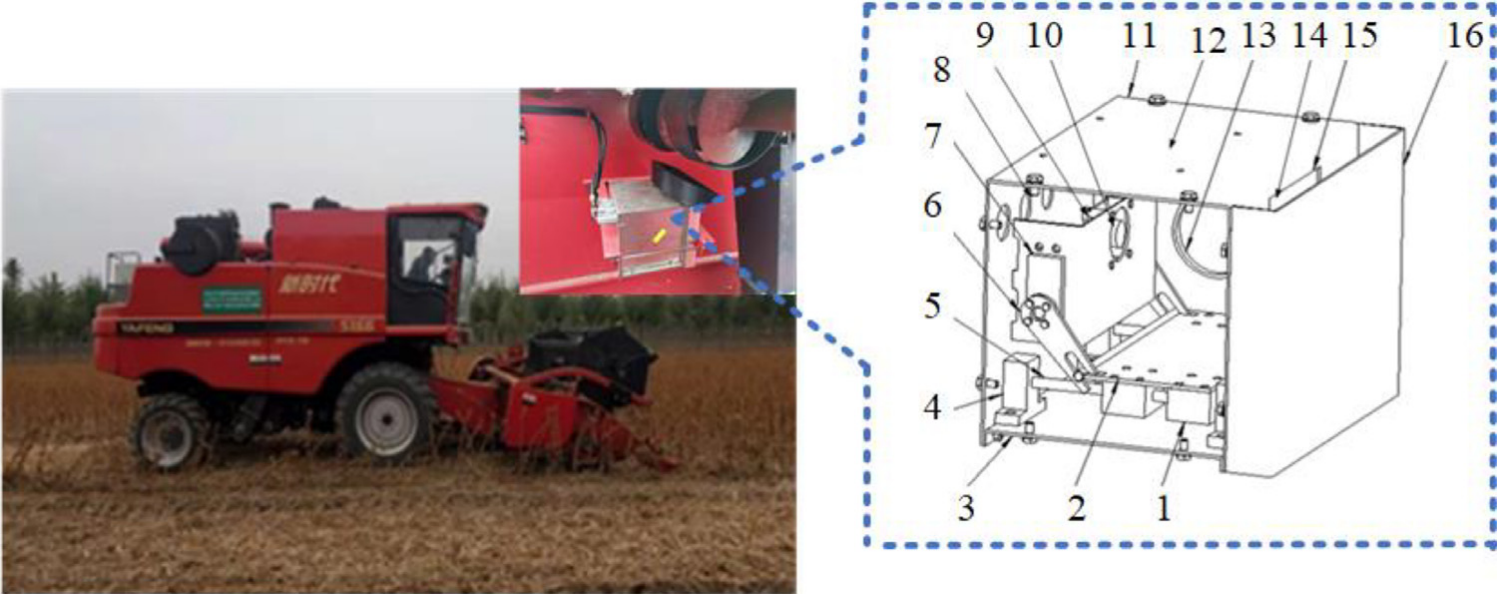
Schematic diagram of image acquisition system. 1. Sliders; 2. telescopic plates; 3. bases; 4. guide rail seats; 5. guide rails; 6. Levers; 7. direct-current steering gears; 8. data bus connectors; 9. industrial cameras; 10. industrial camera fixing bracket; 11. shell; 12. embedded data processing module; 13. light emitting diode visual light source; 14. transparent plexiglass; 15. photo window; 16. sampling chamber.

As shown in Fig. 3, the image acquisition system for mechanized soybean harvesting is mainly composed of an industrial computer, a grain acquisition device, an STM32 lower computer, an industrial camera, an LED light source, an electric motor, and other components [1,2].

The grain collection device is installed below the grain outlet of the soybean combine harvester to collect soybeans as they fall into the grain sampling tank. The industrial control computer collects soybean images through an industrial camera and performs image processing, recognition, and display. The lower computer controls the motor action to obtain real-time mechanized soybean harvest samples, thereby achieving online monitoring of operation quality [3,4].

### 3.2 Experimental design of image acquisition for mechanized harvesting of soybeans

The image collection work was completed on October 9, 2018, in an experimental field of Guanyi, Liangshan, Shandong, China. The soybean variety Zhengdou1307 was planted in the experimental field. During mechanized harvesting, the average moisture content of soybean seeds was 12 %, and the weight of 100 seeds was 22.52 g. The experimental process used a 4LZ-6 intelligent soybean combine harvester (Yafeng Agricultural Machinery, Zibo, Shandong, China) for mechanized soybean harvesting. The mechanized soybean harvesting image acquisition system described in this article was used to collect real-time soybean images in the grain bin during the operation of the soybean harvester.

**Fig. 4:**
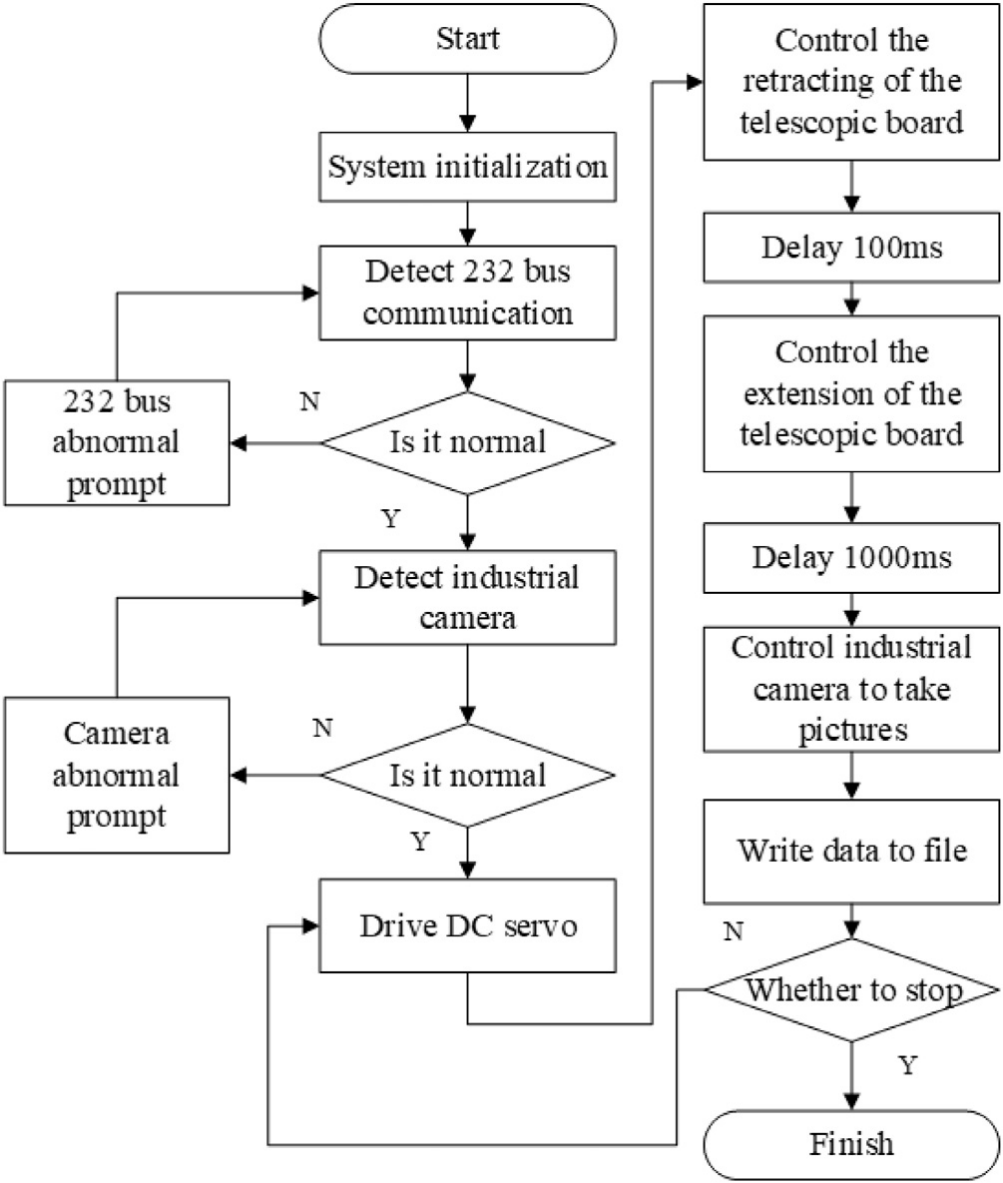
flowchart of image acquisition for mechanized soybean harvesting.

The workflow of the mechanized soybean harvesting image acquisition system is shown in Fig. 4. After powering on the system, perform a self-check to complete the system's initialization settings. Check whether the 232-bus communication and industrial camera are normal, and then output corresponding prompt information if they are not normal. If all working components are working properly, continue to execute the program. The computer sends a DC servo control command and controls the DC servo to drive the expansion plate to retract, and the soybean sample sampling bin releases the soybean sample, with a delay of 100 ms. Send a DC servo control command, control the DC servo to drive the expansion plate to extend, and load the soybean sample in the soybean sample sampling bin with a delay of 1000 ms. Send industrial camera control commands to capture images of soybean samples and save the captured images. After the image is saved, the system controls the action of the DC servo to collect the next soybean sample image until it receives a signal to end the collection task.

### 3.3 Mechanized harvest image annotation and data enhancement

First, we conducted a preliminary screening of the images collected in the field, removing blurry, incomplete, and unusable images [5]. After that, we selected 40 images for constructing the first version of the mechanized harvesting soybean image dataset.

**Fig. 5:**
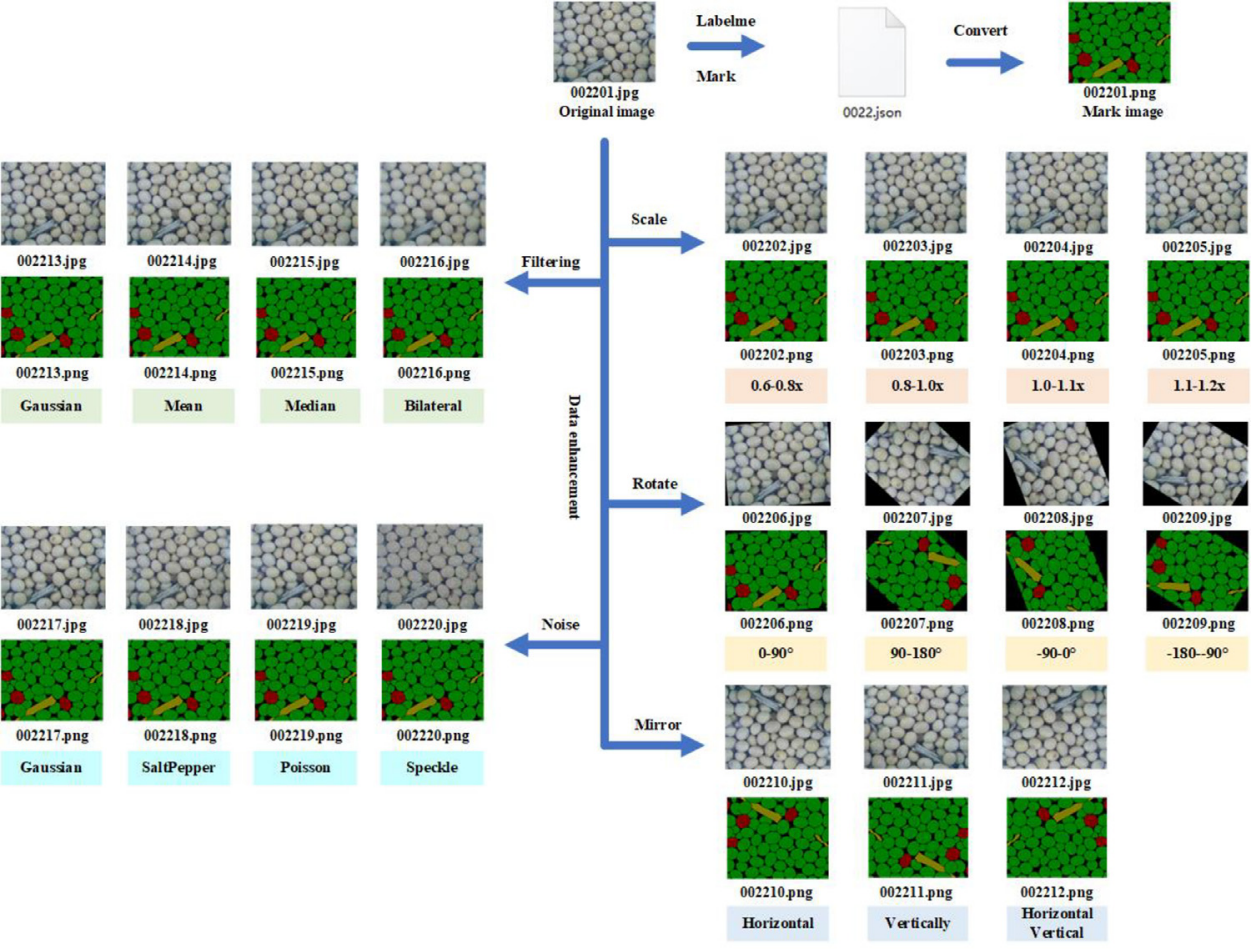
Construction of soybean image dataset for mechanized harvesting.

Next, as shown in Fig. 5, we used the open-source annotation software LabelMe (3.16.7) to annotate the mechanized soybean image dataset [6]. We used polygon annotations to manually label all pomegranates in the collected images as rectangles, labeled as “complete grains,” “broken grains,” “impurities,” and “background,” and we saved the annotation file in JSON format. Afterward, a batch conversion program for annotated images was written in Python language on PyCharm Community Edition. The original images and annotated images were extracted from the JSON file and saved in the JPEGImages and SegmentationClass folders according to naming conventions.

Then, an image data enhancement program was written in Python language on PyCharm Community Edition [7,8], where the image was randomly scaled (0.6–1.2 times), rotated (−180°), mirrored (horizontal, vertical, horizontal vertical), filtered (Gaussian filtering [9,10], mean filtering, median filtering, and bilateral filtering), and subjected to added noise (Gaussian noise, salt and pepper noise, Poisson noise, and Speckle noise). We saved the converted images in the JPEGImages and SegmentationClass folders according to naming conventions.

Finally, the dataset was randomly divided into a training set containing 560 images, a validation set containing 120 images, and a test set containing 120 images.

## 4 Summary and Outlook

The proposed soybean image dataset is designed for the application scenario of mechanized soybean harvesting in the field.

It is intended to be a valuable resource for research on online detection methods and equipment focused on assessing the quality of soybean mechanized harvesting through the use of machine vision and deep learning technology. This dataset provides high-quality images of soybeans with different harvesting qualities, which can be used to study the crushing rate and impurity rate of soybeans during mechanized harvesting operations. This dataset also has the potential to be used for developing machine vision algorithms and deep learning models for automatically estimating the crushing rate and impurity rate of soybean mechanized harvesting operations. Simultaneously, we recognize that the current soybean image dataset is limited in terms of environment and crop variety because it only includes one soybean variety. In addition, the dataset's original image count is relatively small, necessitating further expansion in future research endeavors. Therefore, we plan to expand the soybean image dataset in future research, to include different operating areas, harvesting machines, and soybean varieties, which will increase the diversity, universality, and applicability of the dataset, making it a more effective and valuable resource for research on online detection of soybean mechanized harvesting quality research and the development of related deep learning models. We believe that an expanded soybean image dataset will be beneficial for the research and development of online detection technology for soybean mechanized harvesting quality and will help improve the intelligence of soybean mechanized harvesting.

## Limitations

The core components of the image acquisition system include industrial computer (Model: Xplore X SLATE B10, processor is Intel Core i5 5350 U, graphics card is Intel HD Graphics 6000, system memory is 8GB. Manufacturer: Yibenxuan Technology, Guangzhou, China), STM32 lower computer (CPU: STM32F103ZET6. Manufacturer: Beiyite Technology, Sichuan, China), industrial camera (Model: 1080P (V5610) PCBA, paired with a camera lens with a focal length of 8 mm. Manufacturer: Metro Vision Technology, Guangzhou, China), and servo motor (Model: LX-20S, working voltage 6–8.4 V, rotation range 0–240°, accuracy 0.3° Manufacturer: Fantasy Robot, Sichuan, China).

## Ethics Statement

The proposed data does not involve any human subjects, animal experiments, or data collected from social media platforms.

## CRediT authorship contribution statement

**Man Chen:** Conceptualization, Methodology, Software, Writing – original draft, Investigation. **Chengqian Jin:** Supervision, Validation, Writing – review & editing. **Youliang Ni:** Validation, Writing – review & editing. **Tengxiang Yang:** Validation, Writing – review & editing. **Jinshan Xu:** Validation, Writing – review & editing.

## Data Availability

Mechanical-harvest Soybean Images Dataset (Original data) (Mendeley Data)
